# Non-bacterial Cystitis With Increased Expression of Programmed Cell Death Ligand 1 in the Urothelium: An Unusual Immune-Related Adverse Event After Atezolizumab Administration for Metastatic Breast Cancer

**DOI:** 10.7759/cureus.25486

**Published:** 2022-05-30

**Authors:** Aiko Obayashi, Mai Hamada-Nishimoto, Yuri Fujimoto, Yukiko Yoshimoto, Sachiko Takahara

**Affiliations:** 1 Department of Breast Surgery, Osaka Saiseikai Noe Hospital, Osaka, JPN; 2 Department of Breast Surgery, Osaka Red Cross Hospital, Osaka, JPN; 3 Department of Breast Surgery, Breast Center, Kitano Hospital, Tazuke Kofukai Medical Research Institute, Osaka, JPN

**Keywords:** immune checkpoint inhibitor, breast cancer, case report, cystitis, immune-related adverse events, atezolizumab, anti-pd-l1 antibody

## Abstract

We report a case of non-bacterial cystitis that occurred after administration of atezolizumab, an antibody against programmed cell death ligand 1 (PD-L1). This cystitis was considered an immune-related adverse event (irAE). A 67-year-old woman with advanced breast cancer (cT4bN1M1, cStage IV) was treated with atezolizumab and nanoparticle albumin-bound (nab) paclitaxel. She consulted a physician for urethral pain and frequent urination during the fourth cycle of treatment. Cystitis symptoms were not relieved by antibiotic treatment and worsened. The results of her urine culture and cytology were negative for malignancy. Cystoscopy showed diffuse redness of the bladder mucosa. A bladder biopsy revealed no evidence of malignancy. Since the patient’s symptoms resolved with steroid therapy, urethral pain and frequent urination associated with atezolizumab were considered to be irAE by the diagnosis of exclusion. After immunostaining of the bladder biopsy sections, high PD-L1 expression was detected in the urothelium, which could explain the cause of irAE.

## Introduction

Immune checkpoint inhibitors (ICIs) are increasingly used as therapeutic drugs for various tumors. The immune-related adverse events (irAEs) of checkpoint inhibitors differ from those of conventional chemotherapy [1]. Although various events can occur throughout the body, irAEs affecting the bladder and urinary tract are rare; to date, there is only one case of cystitis associated with atezolizumab therapy [2]. Herein, we report a rare case of cystitis that developed after administration of atezolizumab, an antibody against programmed cell death ligand 1 (PD-L1), in a patient with breast cancer.

## Case presentation

**Figure 1:**
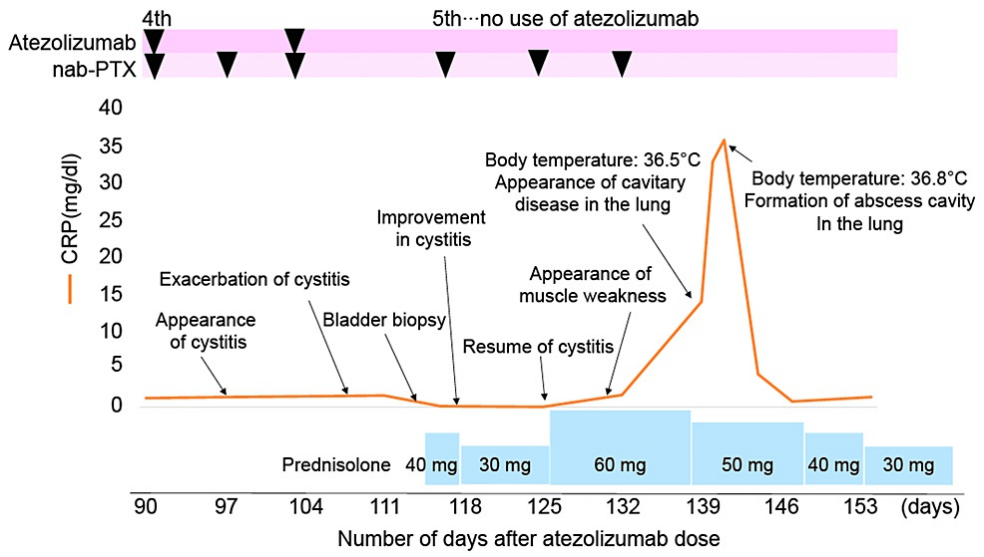
Clinical course of the patient. Although pneumonia was observed on day 139, the patient was asymptomatic without fever production. CRP, C-reactive protein; nab-PTX, nanoparticle albumin-bound paclitaxel.

A 67-year-old woman was diagnosed with advanced breast cancer (mucinous carcinoma, histological grade I, estrogen receptor (ER) (−), progesterone receptor (PgR) (−), human epidermal growth factor receptor 2 (HER2) (1+), Ki-67 (20%), PD-L1 (IC1), cT4bN1M1 (PUL), and cStage IV). She was treated with atezolizumab at a fixed dose of 840 mg on days one and 15 and nanoparticle albumin-bound (nab) paclitaxel at a dose of 100 mg/m^2^ body surface area on days one, eight, and 15 of each 28-day cycle. The patient underwent surgery for bladder cancer (transurethral resection of bladder tumor) at age 56 and experienced no recurrence. She had no history of allergies. The patient’s clinical course is shown in Figure 1.

**Figure 2:**
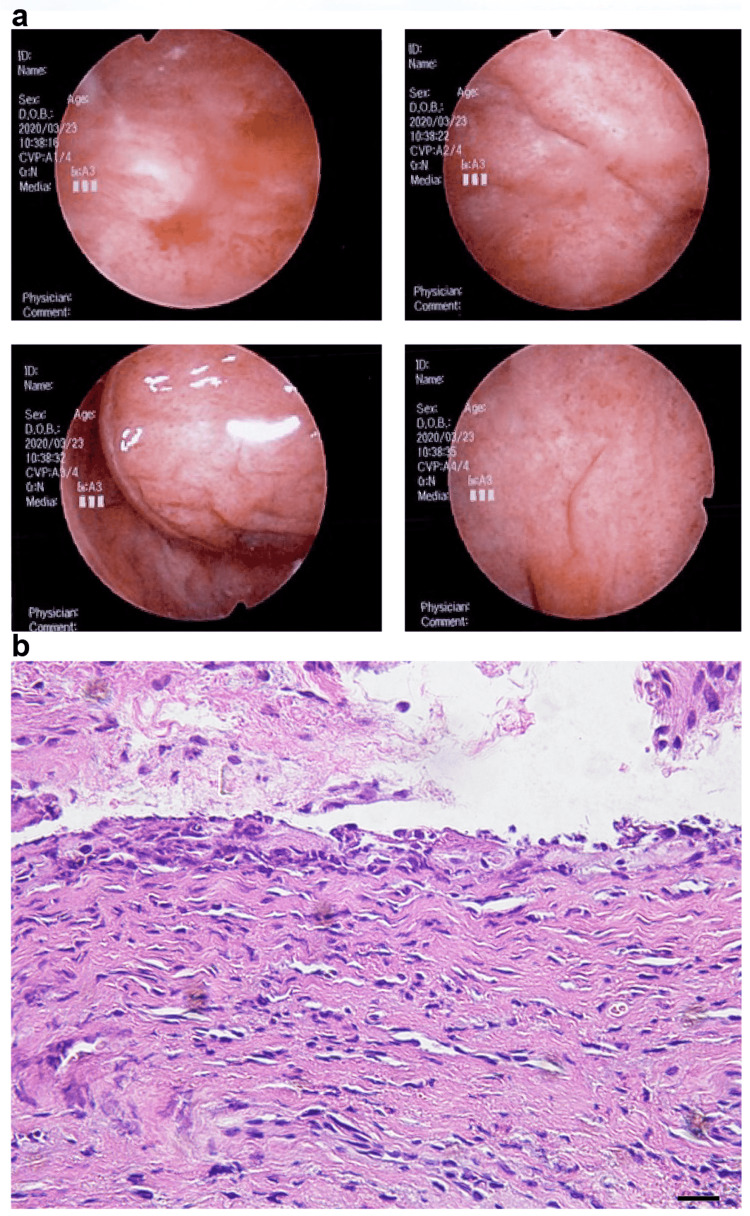
Cystoscopy with bladder biopsy. A: Cystoscopy showed diffuse redness of the bladder mucosa. B: Histopathological examination showed no evidence of malignancy and the absence of inclusion bodies in the epithelium.

Severe urinary tract pain (Common Terminology Criteria for Adverse Events (CTCAE) grade 2) and frequent urination (CTCAE grade 2) developed on day 97 after chemotherapy was started. Although she was treated with trimethoprim-sulfamethoxazole antibiotics, her symptoms did not improve, and her urine culture was negative. Moreover, she could not sleep due to pain in the urethra (CTCAE grade 3). She was admitted to the hospital for a detailed examination. Her urine cytology was negative for malignant cells. Cystoscopy showed diffuse redness of the bladder mucosa (Figure 2A). A bladder biopsy was performed on day 112. Histopathologic examination showed no evidence of malignancy and the absence of inclusion bodies in the epithelium (Figure 2B). Only monocytic and eosinophilic infiltrations were observed in the interstitium.

**Figure 3:**
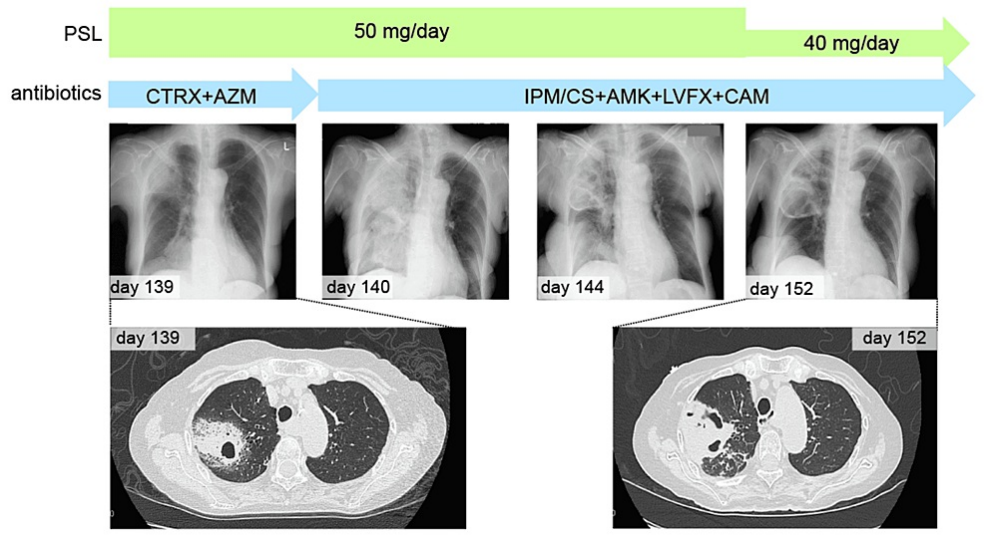
Chest X-ray and CT findings. Cavitary disease in the right lung persisted during antibiotic therapy. PSL, prednisolone; CTRX, ceftriaxone; AZM, azithromycin; IPM/CS, imipenem/cilastatin; AMK, amikacin; LVFX, levofloxacin; CAM, clarithromycin.

Since symptomatic treatment was complex, she started steroid therapy (prednisolone (PSL) at a dose of 40 mg/day (1 mg/kg)) on day 113. Urethral pain and pollakiuria disappeared two days after initiating steroid therapy. PSL was reduced to 30 mg/day on day 117. However, these symptoms recurred on day 125 (CTCAE grade 2). Therefore, PSL was increased to 60 mg/day the same day. On day 132, she developed muscle weakness in her lower extremities and was hospitalized again. Several investigations including blood tests, magnetic resonance imaging, and electromyography did not reveal the cause. As for cystitis, the PSL dose gradually decreased after day 138, but the urethral pain persisted (CTCAE grade 2). Although she had no evidence of infection, continuous blood tests on day 139 showed abnormal C-reactive protein (CRP) and white blood cell counts. On CT, the cavitary disease was detected in the right lung (Figure 3), and a sputum culture showed *Pseudomonas aeruginosa*, *Candida* species, and *Mycobacterium abscessus* on day 145. Despite treatment with several antibiotics, her respiratory condition gradually deteriorated, and she died on day 164.

**Figure 4:**
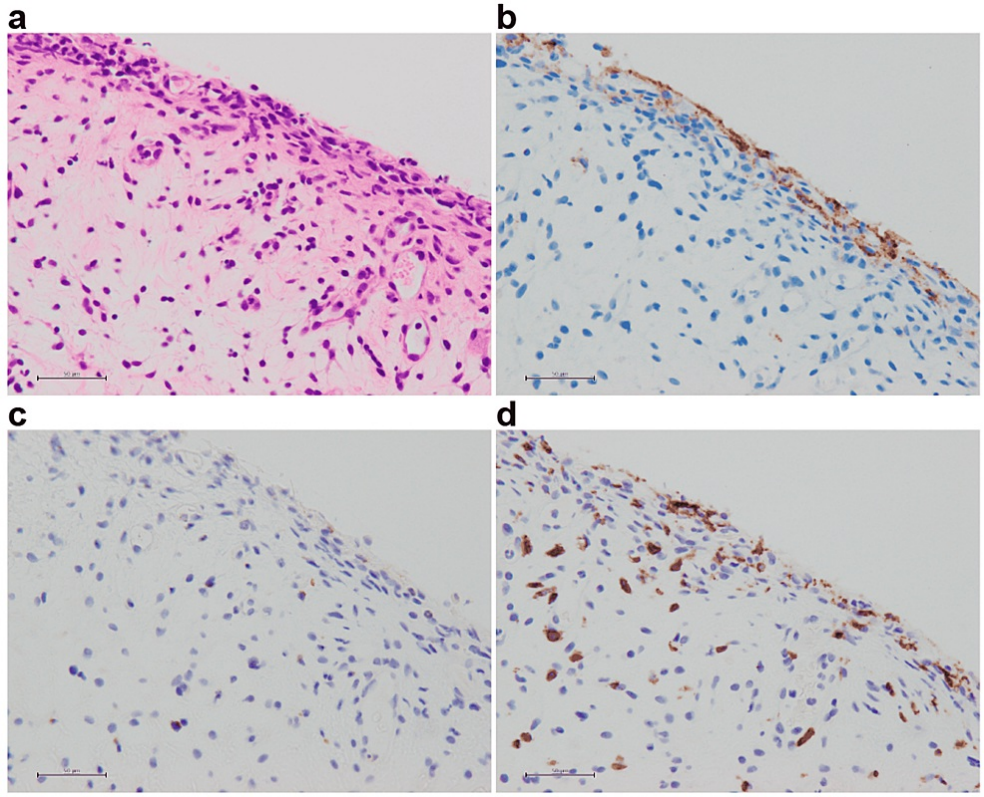
Histopathological image of the urinary bladder. A: HE. B: PD-L1. C: TIA-1. D: CD8. Bar = 50µm. The urothelium strongly expressed PD-L1. Infiltrates of CD8-positive and/or TIA-1-positive lymphocytes are present in the bladder tissue. HE, hematoxylin and eosin; PD-L1, programmed cell death ligand 1; TIA-1, T-cell intracellular antigen 1.

Another pathological examination was conducted after her death. Histological analysis of the urothelium revealed strong PD-L1 expression (stained with SP142), as well as infiltrates of CD8-positive cells and/or T-cell intracellular antigen 1 (TIA-1) (or TIA-1 cytotoxic granule-associated RNA-binding protein)-positive lymphocytes (Figure 4). From the clinical course, the symptom of cystitis was suspected to be an irAE; the histological results were pathologically consistent.

## Discussion

Atezolizumab, an anti-PD-L1 antibody and ICI, is used for lung cancer, hepatocellular carcinoma, and breast cancer. IrAEs caused by ICIs can affect multiple organs and are most commonly observed in the skin, GI tract, lungs, endocrine glands, thyroid gland, adrenal gland, pituitary gland, and musculoskeletal system [1,3]. However, irAEs affecting the bladder and urinary tract are very rarely reported. Four cases of cystitis have been associated with nivolumab [4-6], one case with pembrolizumab [7], and one case with sintilimab [8]. In one case, combined therapy of nivolumab and ipilimumab was used [9]; in one case, cystitis occurred during nivolumab therapy and recurred during atezolizumab therapy [2].

In the present case, the symptoms of cystitis were relieved by PSL rather than antibiotic therapy. In addition, a biopsy of the bladder showed no evidence of malignancy. Therefore, this case of cystitis was diagnosed as irAE. Currently, the diagnosis of cystitis as irAE is made by the diagnosis of exclusion. Steroids are used for diagnostic treatment. However, the risk of side effects associated with steroid administration and the rapid initiation of therapy require specific diagnostic methods for irAE cystitis.

A previous report indicated the presence of PD-L1 expression, after immunostaining of a bladder biopsy, in a case of cystitis believed to be due to irAE [7]. In our case, the expression of PD-L1 in the bladder biopsy was also examined and positively identified. It is possible that atezolizumab acts on PD-L1 in the bladder epithelium and causes cystitis. On the other hand, one case was reported as irAE in which PD-L1 expression was not observed in the bladder epithelium of cystitis [6]. Tables 1, 2 are summaries of case reports of irAE cystitis.

**Table 1:** Information about ICIs and immunostaining. ^a^ Cystitis occurred during nivolumab therapy and recurred during atezolizumab therapy. ^b^ Cystitis occurred during maintenance immunotherapy with nivolumab alone. ^c^ Cystitis occurred during nivolumab therapy. ^d^ Cystitis occurred during atezolizumab therapy. ICI, immune checkpoint inhibitor; SCLC, small cell lung cancer; NSCLC, non-small cell lung cancer; ICC, intrahepatic cholangiocarcinoma; N/A, not available; CR, complete response; PR, partial response; PD-L1, programmed cell death ligand 1; PD-1, programmed cell death protein 1.

Case	Age	Gender	Malignancy	ICI	Best response	ICI cycles before the onset	Examination by immunostaining of bladder biopsy	Reference
1	67	F	Breast cancer	Atezolizumab	PR	4	PD-L1 (+), TIA-1 (+), CD8 (+)	Our case
2	48	M	ICC	Nivolumab → atezolizumab^a^	N/A	3^c^/3^d^	N/A	[2]
3	62	M	NSCLC	Nivolumab	N/A	3	N/A	[4]
4	50	M	NSCLC	Nivolumab	N/A	7	N/A	[5]
5	60	M	NSCLC	Nivolumab	N/A	12	N/A	[5]
6	51	M	SCLC	Nivolumab	CR	5	PD-L1 (−), PD-1 (<1%), CD3 (+), CD4 (1%), CD8 (+), CD19 (1%), CD56 (+)	[6]
7	78	F	NSCLC	Pembrolizumab	PR	6	PD-L1 (+), TIA-1 (+), CD8 (+)	[7]
8	53	M	NSCLC	Sintilimab	PR	3	N/A	[8]
9	61	F	Melanoma	Nivolumab (+ ipilimumab)^b^	PR	4 (nivolumab alone)	CD3 (+), PD-1 (+), CD20 (+)	[9]

**Table 2:** Clinical information on irAE cystitis. ^a^ Cystitis occurred during nivolumab therapy and recurred during atezolizumab therapy. ^b^ Cystitis occurred during maintenance immunotherapy with nivolumab alone. ^c^ Cystitis occurred during nivolumab therapy. ^d^ Cystitis occurred during atezolizumab therapy. irAE, immune-related adverse events; CTCAE, Common Terminology Criteria for Adverse Events; ICI, immune checkpoint inhibitor; N/A, not available; PSL, prednisolone; mPSL, methylprednisolone.

Case	Clinical symptoms	CTCAE grade	Treatment of cystitis	Result	ICI resume
1	Urinary tract pain	G3	PSL 40 mg/day (1 mg/kg) → 30 mg/day → recurred → 60 mg/day	Not improved	No
2	Urinary tract pain^c^/bladder irritation^d^	N/A	ICI discontinuation^c^/steroid hormones (2 mg/kg/day)^d^	Improved	No
3	Urinary tract pain, hematuria	G3	mPSL 500 mg, 3 days → PSL 0.5 mg/kg/day	Improved	Yes
4	Pollakisuria, micturition pain, diarrhea	G3	PSL 1 mg/kg/day	Not improved	No
5	Pollakisuria, dysuria, diarrhea	N/A	ICI discontinuation	Improved	No
6	Frequent urination	G2	mPSL 80 mg twice daily	Improved	No
7	Pollakisuria, nocturia, micturition pain	N/A	PSL 25 mg/day	Improved	No
8	Hematuria, pollakiuria, micturition pain	N/A	mPSL 1 mg/kg/day	Improved	No
9	Pollakiuria, bladder pain, urinary urgency, nocturia, diarrhea	N/A	PSL 0.5 mg/kg/day	Improved	Yes (only nivolumab)

There are four cases, including this case, in which bladder biopsy tissue was immunostained. Of these, three cases examined PD-L1 (two positive and one negative), three cases examined CD8 (all positive), and two cases examined CD3 (both positive). These results are reasonable because the findings suggest that T cells are involved in irAE-induced cystitis.

In another case, it has been reported that the stronger the inflammation, the stronger the expression of PD-L1 in the case of interstitial cystitis [10]. Based on these facts, we considered that PD-L1 is involved in two ways in irAE cystitis. First, it is possible that something triggered an increase in PD-L1 expression in the bladder epithelium, and ICI acted on it. Second, ICI may have caused cystitis and increased the expression of PD-L1 as a result of the inflammation.

Although a case of irAE cystitis due to atezolizumab has been reported, our case could be reported as a pure side effect of atezolizumab because it was used as the first therapy. In addition, our case is the first report of irAE cystitis in breast cancer. Steroids are an important treatment for irAE, but they can be fatal in immunocompromised patients due to the side effects. Currently, the diagnosis of irAE is made by exclusion. Still, if a definitive diagnosis of irAE cystitis is established, unnecessary steroid administration can be avoided, and quick intervention can be possible if necessary. We believe that collecting cases of irAE cystitis in the literature will help to make a definitive diagnosis, which will ultimately lead to the elucidation of the mechanisms underlying this irAE.

## Conclusions

We reported a case of refractory cystitis during the treatment with atezolizumab for breast cancer. This cystitis was supposed to be a rare irAE, due to steroid sensitivity and exclusion of other diseases. Although the mechanism underlying irAEs is still unclear, the immunostaining of the diseased tissue, especially PD-L1 expression, would be an important key to diagnosing irAE.
